# Supplementary material for: Physical Changes of Preschool Children during COVID-19 School Closures in Fujian, China
Source: Int J Environ Res Public Health. 2022 Oct 21;19(20):13699. doi: 10.3390/ijerph192013699 (PMC9602639; doi:10.3390/ijerph192013699)
Supplement: Supplementary file 1 [file ijerph-19-13699-s001.zip › ijerph-1929489-supplementary.pdf]

Table S1 The basic situation of investigations.

City	Total Preschool Children *	Investigations		
		Male	Female	Total *n*(%)
Quanzhou	376256	4580	3507	8087 (27.4)
Fuzhou	282283	3211	2877	6088 (20.6)
Zhangzhou	155719	1726	1550	3276 (11.1)
Longyan	141226	1677	1381	3058 (10.4)
Putian	125473	1417	1233	2650 (9.0)
Ningde	116673	1302	1150	2452 (8.3)
Sanming	89113	1035	921	1951 (6.6)
Nanping	85210	1061	890	1956 (6.6)
Total	1371953	16009	13509	29518

*. Data comes from Fujian Provincial Maternity and Child Information System.

Table S2. Basic characteristics of participating children evaluated in the three periods.

Period	Baseline measured time point	Last measured time point	N	Age 3	4	5	6
After the COVID-19 related school closures	2019.9-11	2020.9-11	1688	332	435	706	215
The same period last year	2018.9-11	2019.9-11	4540	1421	1307	1143	669

Table S3. Overweight and obesity rates among preschool children of different regions in four age groups of Fujian, China.

Age	Children with Overweight		χ^2	*P* *	Children with Obesity		χ^2	*P* *
	Urban	Suburban			Urban	Suburban		
3~	239_a (7.5)	249_a (7.7)	0.143	0.706	138_a (4.3)	144_a (4.5)	0.088	0.767
4~	289_a (7.5)	253_a (7.8)	0.229	0.632	147_a (3.8)	143_a (4.4)	1.593	0.207
5~	505_b (11.5)	465_b (11.2)	0.191	0.662	357_b (8.1)	334_b (8.0)	0.021	0.885
6~7	504_c (14.9)	518_b (12.7)	7.904	**0.005**	310_b (9.2)	366_b (9.0)	0.111	0.739
Total	1537 (10.4)	1485 (10.1)	0.600	0.439	952 (6.4)	987 (6.7)	0.997	0.318
χ^2	143.558	73.684			130.309	97.401		
P #	**<0.001**	**<0.001**			**<0.001**	**<0.001**		

*. Comparison of overweight and obesity rates of different regions.

#. Comparison of overweight and obesity rates in four age groups.

a. b. c: The overweight or obesity proportions found significant of four age groups after applying bonferroni correction are indicated by different letters. （$P<0.05$）.

Table S4. Overweight and obesity rates among preschool children of different genders in four age groups of Fujian, China.

**Age**	**Children with Overweight**			χ^2	***P*** *	**Children with Obesity**			χ^2	***P*** *
	**Male**	**Female**	**Total**			**Male**	**Female**	**Total**		
3~	300$_{a}$ (8.5)	188$_{a}$ (6.5)	488$_{a}$ (7.6)	9.733	**0.002**	167$_{a}$ (4.8)	115$_{a,b}$ (4.0)	282$_{a}$ (4.4)	2.412	0.120
4~	334$_{a}$ (8.9)	208$_{a}$ (6.3)	542$_{a}$ (7.7)	16.553	**<0.001**	186$_{a}$ (4.9)	104$_{b}$ (3.1)	290$_{a}$ (4.1)	14.414	**<0.001**
5~	602$_{b}$ (12.8)	368$_{b}$ (9.5)	970$_{b}$ (11.3)	23.436	**<0.001**	491$_{b}$ (10.5)	200$_{a}$ (5.2)	691$_{b}$ (8.1)	80.417	**<0.001**
6~7	600$_{c}$ (14.9)	422$_{c}$ (12.3)	1022$_{c}$ (13.7)	10.035	**0.002**	442$_{b}$ (10.9)	234$_{c}$ (6.8)	676$_{b}$ (9.1)	37.978	**<0.001**
Total	1836 (11.5)	1186 (8.8)	3022 (10.2)	57.716	**<0.001**	1286 (8.0)	653 (4.8)	1939 (6.6)	122.245	**<0.001**
χ^2	109.148	101.428	207.991			184.038	56.155	226.791		
P #	<0.001	<0.001	<0.001			<0.001	<0.001	<0.001		

*. Comparison of overweight and obesity rates of different sexes.

#. Comparison of overweight and obesity rates in four age groups.

a. b. c: The overweight or obesity proportions found significant of four age groups after applying bonferroni correction are indicated by different letters. (P<0.05）.

Table S5. Baseline characteristics of physical status of participating children during the pre-pandemic period.

Variable	Baseline of Age 4 group (Age 3 at the time)		t/χ^2	P	Baseline of Age 5 group (Age 4 at the time)		t/χ^2	P	Baseline of Age 6 group (Age 5 at the time)		t/χ^2	P
	2018 (n=1307)	2019 (n=435)			2018 (n=1143)	2019 (n=706)			2018 (n=669)	2019 (n=215)		
Height, cm	101.09±4.48	100.83±4.66	1.020	0.308	105.70±5.87	106.14±4.90	-1.757	0.079	110.12±5.16	109.11±6.21	2.157	**0.032**
Weight, kg	16.03±2.24	15.88±2.02	1.233	0.218	17.32±2.75	17.58±2.82	-1.907	0.057	18.52±2.80	18.74±3.86	-0.760	0.448
BMI	15.63±1.44	15.58±1.30	0.696	0.487	15.43±1.42	15.53±1.63	-1.330	0.184	15.21±1.46	15.60±1.89	-2.738	**0.007**
People with overweight	175(13.4)	62(14.3)	0.709	0.702	129(11.3)	85(12.0)	2.443	0.295	62(9.3)	23(10.7)	5.780	0.056
People with obesity	59(4.5)	16(3.7)			48(4.2)	40(5.7)			27(4.0)	17(7.9)		
